# Frequency-Domain Optical Coherence Tomography for Intracranial Atherosclerotic Stenosis: Feasibility, Safety, and Preliminary Experience

**DOI:** 10.3389/fneur.2021.678443

**Published:** 2021-06-17

**Authors:** Bin Yang, Yiding Feng, Yan Ma, Yabing Wang, Jian Chen, Long Li, Jia Dong, Bairu Zhang, Peng Gao, Yanfei Chen, Adam A. Dmytriw, Liqun Jiao

**Affiliations:** ^1^Department of Neurosurgery, Xuanwu Hospital, Capital Medical University, Beijing, China; ^2^Department of Interventional Neuroradiology, Xuanwu Hospital, Capital Medical University, Beijing, China; ^3^Neuroradiology & Neurointervention Service, Brigham and Women's Hospital and Harvard Medical School, Boston, MA, United States

**Keywords:** intracranial stenosis, ischemic stroke, optical coherence tomography, percutaneous intervention, vertebrobasilar insufficiency

## Abstract

**Background:** Despite advances in non-invasive imaging, the characterization of atherosclerotic plaque remains superior with frequency-domain optical coherence tomography (FD-OCT) in the clinical coronary and experimental cerebrovascular literature. An assessment of the feasibility and safety of FD-OCT for intracranial atherosclerotic stenosis (ICAS) is desirable.

**Methods:** We analyzed a cohort of all consecutive FD-OCT evaluations for ICAS performed at our institution from April 2017 to August 2018 (16 months) in patients who suffered from transient ischemic attack (TIA) or non-disabling stroke despite optimal medical management within 90 days of admission attributable to angiographically verified 70–99% stenosis of an intracranial artery.

**Results:** Thirty-three patients harboring 36 lesions with an average age of (57.6 ± 7.1) years (male sex 27 cases) comprising nine cases of lesions located within the anterior circulation and 24 cases within the posterior circulation were identified. Of the 33 patients with 36 lesions, the FD-OCT imaging catheter detected 35/36 (97%) lesions except in one case in which the FD-OCT catheter failed to navigate excessively tortuous vessels, and FD-OCT images in 27 patients (81.8%) were finally obtained successful, where the target lesion was fully visible, and image quality under at least one pullback was graded 2 or 3. There were no symptomatic complications. Blood flow was the most common artifact encountered (51.9%).

**Conclusion:** FD-OCT is safe and feasible for the assessment of ICAS in the anterior and posterior circulation. The use of diagnostic interferometry will have to be weighed against its cost, and these preliminary findings should be verified by prospective large-scale studies.

## Introduction

Intracranial atherosclerotic stenosis (ICAS) is a major worldwide cause of stroke, accounting for 30–50% and 8–9% ischemic events in Asians and Caucasians, respectively (1, 2). Its presence is accurately diagnosed by conventional imaging modalities including computed tomography angiography (CTA), magnetic resonance angiography (MRA), and digital subtraction angiography (DSA). However, these methods are insufficient for the characterization of plaque composition. Novel non-invasive methods have achieved some success in this domain, such as high-resolution vessel wall magnetic resonance imaging (HR VW-MRI), which can depict morphology and determine the location of plaque relative to branch artery ostia. However, even its sub-millimeter resolution (typically <0.7 mm) does not always completely satisfy the clinical need for plaque characterization (3–6).

Frequency-domain optical coherence tomography (FD-OCT) is a relatively new intravascular imaging modality in the neuroendovascular armamentarium with ultra-high resolution (approximately 10 μm), which can offer expanded spatial detail of plaque characteristics. The scope of FD-OCT has extended from the coronary arteries to the peripheral and cerebrovascular circulation within the last 10 years (7–13). Of late, there have been several reports regarding its application within intracranial arteries in carefully selected patients (14–19). However, the data surrounding feasibility and safety in the context of ICAS assessment are still limited. In this study, we report our preliminary experience of FD-OCT in the largest cohort of ICAS patients of which we are aware of.

## Methods

### Patient Selection and Study Design

We analyzed a prospective cohort of all consecutive FD-OCT evaluations for ICAS performed at our institution from April 2017 to August 2018 (16 months). These patients all suffered from transient ischemic attack (TIA) or non-disabling stroke despite optimal medical management within 90 days of admission. Ischemic events were attributable to angiographically verified 70–99% stenosis of an intracranial artery based on Warfarin-Aspirin Symptomatic Intracranial Disease (WASID) criteria (20). Percutaneous transluminal angioplasty and stenting (PTAS) was performed with a balloon-mounted stent or self-expandable stent. Clinical data including patient age, gender, National Institutes of Health stroke scale (NIHSS), serum creatinine, Mori type, and operative time were recorded. The study protocol was approved by our ethics committee, and written informed consent was obtained from all patients. The study was approved our Research Ethics Board.

### Frequency-Domain Optical Coherence Tomography Techniques in Intracranial Atherosclerotic Stenosis

FD-OCT images were obtained with the Ilumien Optis System (St. Jude Medical, St. Paul, MN, USA) employing a non-occlusive technique. The procedure was performed under general anesthesia. A 6F Navien distal intracranial catheter (Medtronic, Irvine, CA, USA) with an 8F ENVOY guiding catheter (Codman Neurovascular, Raynham, MA, USA) was advanced to the distal part of V2 segment of the vertebral artery or C1 of the carotid artery *via* transfemoral approach. Then, a 0.014-in. Transcend microwire (Stryker, Kalamazoo, MI, USA) was introduced into the vessel and passed through the site of stenosis with the guidance of an Echelon-10 microcatheter (Medtronic, Minneapolis, MN, USA). The tip of the microwire was navigated to either the P2 segment of posterior cerebral artery (PCA) for posterior circulation cases or M2 segment of middle cerebral artery (MCA) for anterior circulation cases. The Echelon microcatheter was exchanged for a 2.7F Dragonfly Duo OCT catheter (St. Jude Medical, St. Paul, MN, USA), and the imaging catheter was positioned distal to the index region of interest using a standard “monorail” approach. The catheter was used at two different scanning ranges, 75 and 54 mm. The 75-mm scanning length was employed for longer lesions, the higher-detail 54-mm range was chosen by default, and the pullback speed was set to 18 mm/s.

OCT acquisition was performed using the automatic injection system (Mark V ProVis; Medrad Interventional/Possis, Warrendale, PA, USA) in the first 20 patients. Nine milliliters of iso-osmolar Visipaque contrast medium (GE Healthcare, Cork, Ireland) was injected through an Navien catheter at a rate of 3 ml/s at 200 psi. Acquisition was achieved by hand injection of the same volume contrast medium in the last 13 patients. Pullback was trigged as soon as the blood was completely replaced by the contrast medium distal to the interested region. During image acquisition, continuous images were stored digitally for subsequent analysis.

### Frequency-Domain Optical Coherence Tomography Image Analysis

The image quality of all FD-OCT pullbacks was assessed by two experienced investigators (LL and BY), graded on a predefined four-category scale as proposed in the coronary literature (21). If <10% of cross sections in a pullback were considered suitable to be analyzed, the image quality was defined as grade 0; if 10–50% of cross sections in a pullback were suitable, the image quality was defined as grade 1; if 51–90% of cross sections in a pullback were suitable, the image quality was defined as grade 2; and if >90% of cross sections in a pullback were suitable, the image quality was defined as grade 3.

Examination was considered successful when (1) image acquisition could be adequately performed, (2) the target lesion was fully visible, and (3) the image quality under at least one pullback was graded 2 or 3. Examination was considered unsuccessful if (1) image acquisition could not be performed due to technical or anatomical problems, or (2) the target lesion could not be reliably assessed, or (3) image quality in all pullbacks was graded 0. Examination was considered partially successful if image quality under at least one pullback was graded 1, and at least a part of the region of interest could be assessed.

### Artifact Definition

**Figure 1:**
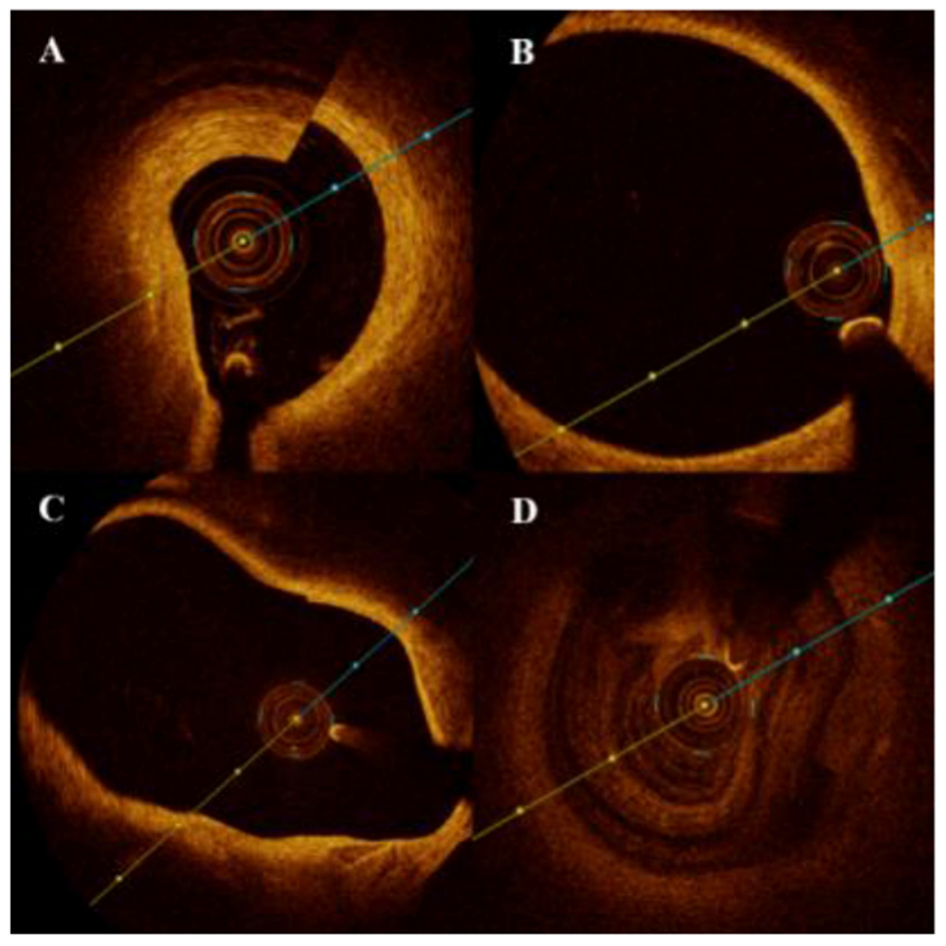
Artifacts of optic coherence tomography for the detection of intracranial atherosclerotic stenosis (ICAS). **(A)** Rotational artifact appears as an apparent misalignment of the luminal border due to rapid axial rotation of the imaging catheter during pullback. **(B)** Decentration artifact appears as the distortion and/or invisible part of image because of the eccentric position of frequency-domain optical coherence tomography (FD-OCT) catheter. **(C)** Caliber artifact was defined as the integrity loss of the whole lumen due to the large diameter of the vessel, and **(D)** blood-flow artifact was defined as light dissipation caused by the residual blood due to inadequate replacement.

OCT rotational artifact was defined as an apparent misalignment of the lumen border due to rapid axial rotation of the imaging catheter during pullback. Decentration artifact was defined as the distortion and/or invisible image section due to eccentric position of FD-OCT catheter. Caliber artifact was defined as integrity loss of the whole lumen due to the large diameter of the vessel, and lastly blood artifact was defined as light occlusion caused by residual blood due to inadequate replacement (Figure 1) per standard definitions.

### Tortuosity Index

Tortuosity index was defined as the ratio of the linear length to the real length between the first and third markers of the FD-OCT image catheter. The linear length was measured by the DSA software (Siemens, Munich, Germany). All these data were measured by two independent and experienced neuroradiologists. The average of the two data was the final recorded linear length.

### Safety Evaluation

NIHSS data were obtained before, immediately after, and 24 h after surgery by two independent and experienced neurologists. Blood creatinine was measured before surgery and 24 h after surgery. If postoperative NIHSS was higher than baseline, MRI was performed to confirm ischemic or hemorrhagic events. Acute kidney injury (AKI) was defined as an increase of ≥44.2 μmol/L and/or an increase of ≥30% of preoperative to postoperative blood creatinine levels within 48–72 h after the procedure.

## Results

**Figure 2:**
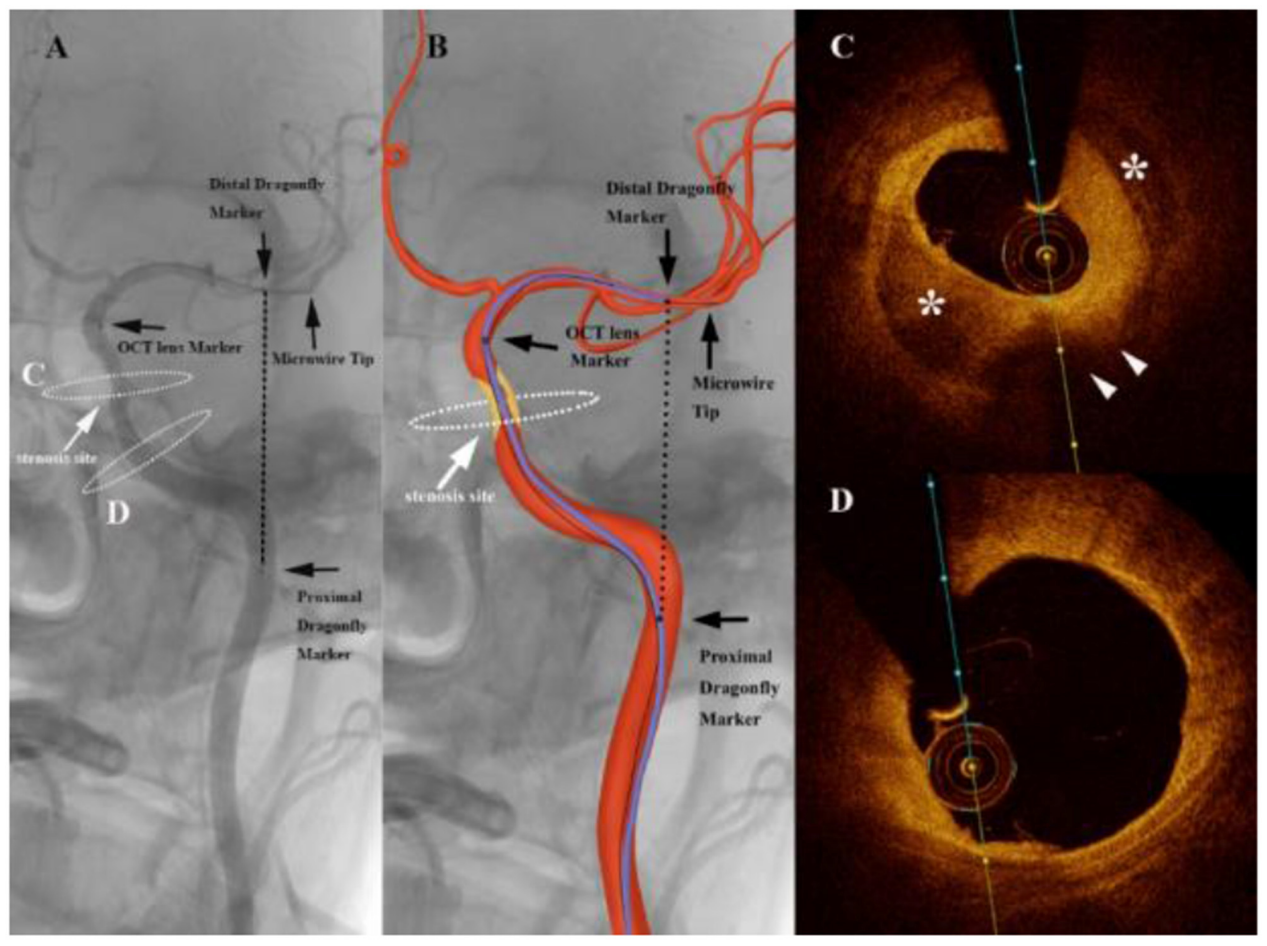
Angiography of an intracranial atherosclerotic stenosis (ICAS) patient suffering from repeated weakness of the right upper limb with dual-antiplatelet therapy **(A)** showing a severe stenosis of the internal carotid artery (white arrows). The graphic of the imaging catheter and artery is overlaid in the middle panel **(B)**. The markers of the Dragonfly Duo imaging catheter and the optical coherence tomography (OCT) lens are noted in the left and middle panels (black arrows). The black dashed line represents the linear length between the first and third markers. White dashed ellipses on the left panel correspond to the cross section of the OCT acquisition. **(C)** Frequency-domain optical coherence tomography (FD-OCT) imaging demonstrated the atherosclerotic plaque (white arrowheads) with calcification (*), which imparted the severe stenosis. **(D)** 10 mm proximal to the cross section A, the artery wall was almost normal.

**Figure 3:**
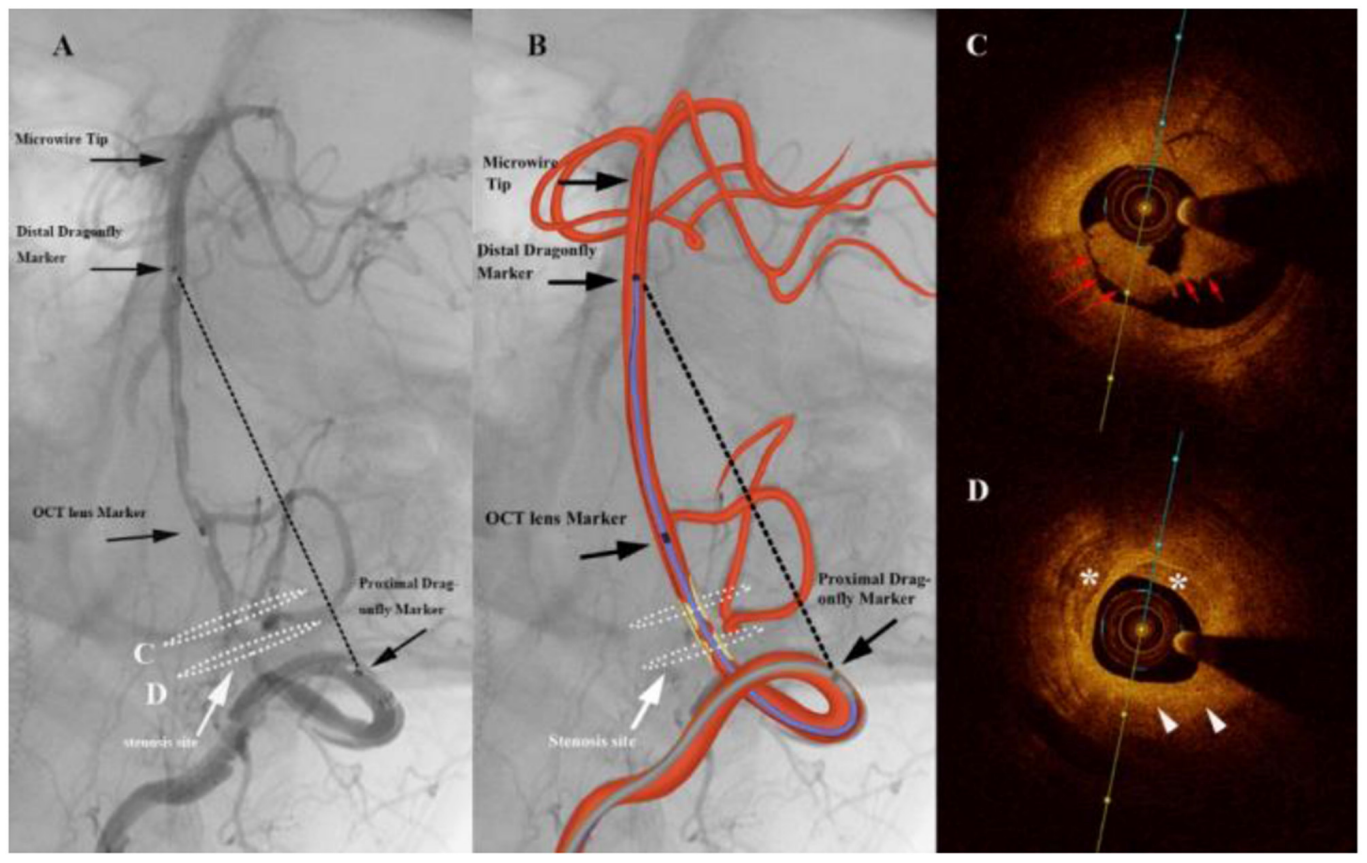
Angiography of a patient complaining of dizziness and slurred speech for 5 months **(A)** shows a severe stenosis of the vertebral artery (white arrows). The graphic of the imaging catheter and artery is overlaid in the middle panel **(B)**. The markers of the Dragonfly Duo imaging catheter and the optical coherence tomography (OCT) lens are noted in the left and middle panels (black arrows). The black dashed line represents the linear length between the first and third markers. White dashed ellipses on the left panel correspond to the cross section of the OCT acquisition. **(C)** Frequency-domain optical coherence tomography (FD-OCT) imaging demonstrated intraluminal thrombosis (red arrows). **(D)** Atherosclerotic plaque (white arrowheads) with calcification (*) were found at the site of stenosis.

A total of 33 patients were included in this study. Baseline data are shown in Table 1. Thirty-three cases of patients with an average age of (57.6 ± 7.1) years (male sex 27 cases) comprising nine cases of lesions located within the anterior circulation (Figure 2) and 24 cases within the posterior circulation (Figure 3) were identified. There were a total of 36 severe stenotic lesions, including 11 cases of Mori type A, 15 cases of type B, and seven cases of type C. The FD-OCT imaging catheter successfully detected 35/36 (97%) lesions except one case in which the FD-OCT catheter failed to navigate excessively tortuous vessels, related to an ophthalmic segment stenosis. All patients completed FD-OCT pullback 52/52 (100%) times in total, with an average of 1.58 ± 0.74 pullbacks per person. Pullback in the anterior circulation was performed 13 times, with an average of 1.44 times per person, among which the imaging quality was grade 2 six times and level 3 seven times. Pullback in the posterior circulation was performed 39 times, with an average of 1.63 times per person, of which the imaging quality was grade 0 one time, grade 1 six times, grade 2 12 times, and grade 3 19 times. The tortuosity index was 0.74 ± 0.13 on average. The tortuosity index for the posterior circulation was significantly higher than that of anterior circulation (0.78 ± 0.12 vs. 0.65 ± 0.12, *p* = 0.01).

**Table 1:** Baseline clinical and procedural data.

**Variable**	**Total**	**Anterior**	**Posterior**
		**circulation**	**circulation**
Cases	33	9	24
Age (years)	57.6 ± 7.1	60.2 ± 8.6	56.7 ± 6.5
Male gender	27 (81.8)	9 (100)	18 (75)
Pullbacks	52	13	39
Pullbacks per examination	1.58 ± 0.75	1.44 ± 0.53	1.63 ± 0.82
Tortuous index^*^	0.74 ± 0.13	0.65 ± 0.12	0.78 ± 0.12
Stroke	28 (84.8)	7 (77.8)	21 (87.5)
Hypertension	20 (60.6)	7 (77.8)	13 (54.2)
DM	18 (54.5)	6 (66.7)	12 (50)
Hyperlipidemia	8 (24.2)	3 (33.3)	5 (20.8)
CHD	3 (9.1)	1 (11.1)	2 (8.3)
Smoking	17 (51.5)	4 (44.4)	13 (54.2)
Drinking	16 (48.5)	5 (55.6)	11 (45.8)
mRS ≤ 2	32 (96.9)	9 (100)	23 (95.8)
Mori type			
A	12 (36.4)	5 (55.6)	7 (29.2)
B	15 (45.5)	2 (22.2)	13 (54.2)
C	6 (18.2)	2 (22.2)	4 (16.7)
Stenosis (%)	78.1 ± 5.7	77.5 ± 5.4	78.3 ± 6.0
Preoperation Cr (μmol/L)	65.6 ± 13.9	68.1 ± 9.0	63.9 ± 15.5
Postoperation Cr (μmol/L)	70.2 ± 17.8	80 ± 14.6^#^	66.5 ± 17.7

*Continuous variables are presented as mean ± SD, while categorical variables are presented as frequency (%). DM, diabetes mellitus; CHD, coronary heart disease; mRS, modified Rankin Scale; Cr, creatinine*.

* *Student's t-test, p = 0.01*.

# *Student's t-test, p = 0.006*.

From femoral artery puncture to completion of OCT withdrawal, the average time was 36.7 ± 12.4 min. The average amount of contrast agent per patient was 57.8 ± 13.2 ml. NIHSS was consistent before and after procedure in all patients. The average serum creatinine before angiography and intervention was 65.6 ± 13.9 U/L, and the average serum creatinine afterward was 70.2 ± 17.8 U/L. The serum creatinine was significantly higher afterward in patients with a lesion in anterior circulation (Table 2; 80 ± 14.6 vs. 68.1 ± 9.0, *p* = 0.006), but no AKI was observed. Iatrogenic dissection occurred in two patients, but neither transcatheter nor medical intervention was needed in either patient. No death or other severe procedural complications were observed in 7 days post-procedure. All patients had the same post-procedural NIHSS compared with pre-procedure.

**Table 2:** Procedural complications.

	**ICA**	**V4**	**BA**
Examination	9	19	5
Total complications	0	2 (10.5)	0
Death	0 (0.0)	0 (0.0)	0 (0.0)
Iatrogenic dissection	0 (0.0)	2 (10.5)	0 (0.0)
AKI	0 (0.0)	0 (0.0)	0 (0.0)
Myocardial infarction	0 (0.0)	0 (0.0)	0 (0.0)
Major stroke hemorrhages	0 (0.0)	0 (0.0)	0 (0.0)
Major non-stroke hemorrhages	0 (0.0)	0 (0.0)	0 (0.0)

*Variables are presented as frequency (%). ICA, internal carotid artery; V4, intracranial vertebral artery; BA, basilar artery; AKI, acute kidney injury*.

Image quality is shown in Table 3. In all 52 pullbacks, the image quality reached grade 3 26 times (50%), grade 2 18 times (34.6%), grade 1 six times (11.5%), and level 0 two times (3.8%). The grade 0 pullbacks occurred in the first two consecutive patients. Blood artifacts were observed in 27 pullbacks, which was the most common (51.9%), decentration artifacts in 13 pullbacks (25%), caliber artifacts in three pullbacks (5.8%), and rotational artifacts in 10 pullbacks (19.2%). Of the 33 patients, 27 underwent successful FD-OCT (81.8%), three were partially successful (9%), and three were unsuccessful (9%). For the intracranial internal carotid artery, eight cases were successful, and one case was a failure. For intracranial vertebrobasilar artery (V4), 15 cases were successful, two cases were partially successful, and two cases were a failure per the established definitions. For the basilar artery (BA), four cases were successful, and one case was partially successful. No significant difference was found between the examination success rate of the posterior and anterior circulation (91.7 vs. 88.9%, *p* = 1). The tortuosity indices of successful, partially successful, and unsuccessful groups were significantly different (Table 4; 0.715 ± 0.125 vs. 0.846 ± 0.067 vs. 0.890 ± 0.057, *p* = 0.03), but further multiple comparisons (Tukey's test) showed no significant difference between all three groups (i.e., successful, partially successful, and unsuccessful). No correlation was discovered with age, gender, underlying disease status, stenosis degree, or Mori classification.

**Table 3:** Image quality of optical coherence tomography.

**Image quality**	**Total**	**ICA**			**V4 (%)**	**BA (%)**
		**C2~C3**	**C4**	**C6**		
3	26 (50)	5 (62.5)	2 (66.7)	0 (0.0)	15 (51.7)	4 (40)
2	18 (34.6)	3 (37.5)	1 (33.7)	2 (100)	8 (27.6)	4 (40)
1	6 (11.5)	0 (0.0)	0 (0.0)	0 (0.0)	4 (13.8)	2 (20)
0	2 (3.9)	0 (0.0)	0 (0.0)	0 (0.0)	2 (6.9)	0 (0.0)

*Variables are presented as frequency (%). ICA, internal carotid artery; C2, petrous segment; C3, lacerum segment; C4, cavernous segment; C6, ophthalmic segment; V4, vertebral artery V4 segment; BA, basilar artery*.

**Table 4:** Success and tortuosity index of OCT examination.

**Variable**	**Successful**	**Partially successful**	**Failure**
**Lesion position**			
ICA	8 (88.9)	0 (0.0)	1 (11.1)
V4	15 (78.9)	2 (10.5)	2 (10.5)
BA	4 (80)	1 (20)	0 (0.0)
Tortuosity index^*^^#^	0.715 ± 0.125	0.846 ± 0.067	0.889 ± 0.057

*Tortuosity index is presented as mean ± SD, while categorical variables are presented as frequency (%). S, successful; PS, partially successful; F, failure*.

* *One-way ANOVA, p = 0.03*.

# *Tukey's test for multiple comparisons: S–PS p = 0.185, F–S p = 0.056, and F–PS p = 0.893*.

## Discussion

In this study, we performed FD-OCT in ICAS patients with standard non-occlusive technique in what is the largest reported cohort to date. The FD-OCT imaging catheter reached the distal end of the stenosis and completed at least one pullback in all cases. In 2014, Given et al. (15) reported FD-OCT images of Wingspan stent implantation for vertebrobasilar artery stenosis, and subsequently Gao et al. (14) employed FD-OCT to detect basilar artery dissection. Building on these case reports, we performed a safety study of FD-OCT evaluation for ICAS, suggesting feasibility for obtaining high-quality FD-OCT images.

### Feasibility of Frequency-Domain Optical Coherence Tomography for Intracranial Arteries

In 2011, Mathews et al. (22) performed OCT scanning in the cavernous sinus segment and the petrous segment of internal carotid artery for the first time in the literature, which used time-domain OCT assembled in a laboratory rather than a commercial product. Although, a small number of cases were enrolled, the result suggested that OCT examination of intracranial arteries was feasible. OCT was then applied to the field of the treatment of intracranial aneurysms using flow diverters (23–25). These authors considered that there were two difficulties in OCT examination for intracranial arteries. Firstly, blood was difficult to clear completely. Secondly, anatomical tortuosity limited the placement of the OCT catheter.

As red blood cells and near-infrared light interact poorly, circulating blood must be completely cleared to obtain a high-quality image *via* current transcatheter interferometric technology. Initially, OCT was applied in coronary arteries by using a detachable balloon to arrest flow and flushing saline in order to replace blood completely (26). However, with the progress of imaging techniques, non-occlusive methods by continuous injection of contrast media to clear blood can also obtain high-quality images. The new generation of Dragonfly Duo OCT imaging catheters can achieve faster pullback speed and longer pullback distances than previously methods, which helps to reduce contrast agent volumes and improves rotational artifacts (27). In this study, the first two patients presented with intracranial vertebral artery severe stenosis. The 6F guiding catheter was placed in the distal end of V2 segment per routine. Although, the OCT catheter could successfully reach the predetermined position and complete the pullback, severe blood artifacts were observed due to poor blood clearance. In subsequent cases, the Navien distal intracranial catheter was used. Due to its flexibility, the distance between the catheter tip and the target lesion was minimized to enhance blood clearance, and the image quality was significantly improved. Unmistakably, technique contributed to the first scores being low, and a learning curve for OCT implementation was at play.

There was one failed case of tandem severe stenosis of cavernous sinus segment and petrous segment of ICA because tortuosity made the OCT imaging catheter unable to reach a stenosis located at the distal end of the cavernous sinus segment. We investigated the tortuosity index of the artery and found that the tortuosity index of ICA was significantly lower than that of the V4 and BA. Furthermore, a significant difference was observed in the tortuosity indexes among successful, partially successful, and unsuccessful groups. Although, the multiple comparisons did not show a difference between each group, this is likely due to the small number of patients in the series for Tukey's calculation. The overall result suggests the tortuosity index may quantifiably assess the tortuosity of intracranial arteries and might be a potential predictor of success of OCT scanning. Although, there is a 20-mm rapid exchange tip in the front of Dragonfly Duo catheter, the tapered design allows the catheter to pass through arteries more easily in its current iteration. Nevertheless, its initial design for detection of coronary disease does not inherently consider the anatomical features of intracranial arteries.

### Imaging Technology

Yoshimura et al. (28) reported two failed cases due to early withdrawal of the imaging OCT catheter during non-occlusive technique in the carotid artery. To avoid this phenomenon, our strategy was to begin withdrawal as soon as the distal blood clearance was apparent. For some cases, when contrast agent could not pass through a severe stenosis, pullback was initiated 1–2 s after injection. The withdrawal of the Dragonfly Duo OCT imaging catheter in high-resolution mode takes 3 s, so the injection parameters are set at 3 ml/s with a total volume of 9 ml. In particularly, filling the Navien catheter with contrast media before injection could reduce blood interference and improve image quality significantly. The microwire and imaging catheter are appropriately retracted to release tension and maintain coaxial positioning after the OCT imaging catheter passes through the stenotic lesion, which can reduce the decentration artifacts and rotational artifacts.

### Safety of Frequency-Domain Optical Coherence Tomography for Intracranial Artery

Most of the early OCT detection data for intracranial arteries were case reports, and no safety reports have been formally published. Reimers et al. (29) used a proximal balloon occlusion technique for OCT examination in seven patients undergoing carotid artery stenting (CAS), and only one patient could not tolerate occlusion during the operation, although, they recovered completely after recanalization of blood flow. Yoshimura et al. (28) also applied proximal occlusion for OCT detection of 34 cases of carotid artery stenosis, with no perioperative complications reported.

In an OCT study before and after carotid artery stent implantation using non-occlusive technique by Setacci et al. (30), no complications were reported in all 25 patients. In our study, all patients were assessed under general anesthesia. A total of 52 OCT pullbacks were performed in 33 patients, with 9 ml of contrast agent used in each retraction. The serum creatinine level elevated mildly after operation, especially in the anterior circulation cases, but no AKI was observed. We adopted distal intracranial catheter as a carrier of the OCT imaging catheter to obtain better blood clearance. As the distance navigated by the catheter within the blood vessel increases, the likelihood of vascular injury increases. Two cases of V4 segment iatrogenic dissection were confirmed by DSA, and both were caused by the distal intracranial catheter. Fortunately, blood flow was not impeded.

FD-OCT has been a powerful tool in the treatment of atherosclerosis of coronary artery, assessing the plaque characterization and guiding the stent implantation (31, 32). With ultra-high resolution, it can also help to study the plaque morphology and composition in ICAS, such as vasa vasorum (33) and neovascularization (34), which have potential contribution to recurrent ischemic events beyond the mere degree of stenosis. Further, large prospective studies and cost–benefit analyses are needed.

### Limitations

This study is based on a single-center retrospective data, and while the sample size is the largest to date, it is still modest. Regarding the complications of OCT examination, there is no established control group. Lastly, there were more posterior circulation than anterior circulation cases, which may reflect selection bias.

## Conclusion

This study suggests the feasibility and safety of FD-OCT for ICAS using a non-occlusive technique. Diagnostic images are obtained in the majority of cases. Further, large prospective studies and cost–benefit analyses are needed.

## Data Availability Statement

The raw data supporting the conclusions of this article will be made available by the authors, without undue reservation.

## Ethics Statement

The studies involving human participants were reviewed and approved by Capital Medical University. The patients/participants provided their written informed consent to participate in this study.

## Author Contributions

BY initiated the study. YF has been involved in the study design and drafted this manuscript. YM, YW, JC, LL, PG, YC, AD, and LJ have been involved in the conception and study design. JD and BZ has made important statistical contributions. All authors provided feedback on drafts of this paper, read, and approved the final manuscript.

## Conflict of Interest

The authors declare that the research was conducted in the absence of any commercial or financial relationships that could be construed as a potential conflict of interest.
